# Supporting researchers through full-service hotline and consultation services: Success in simplicity, customization, and staffing

**DOI:** 10.1017/cts.2019.428

**Published:** 2020-01-10

**Authors:** Rebecca Namenek Brouwer, Emily Miller, Sunita Patil, Geeta K. Swamy, Rebbecca Moen, Amanda McMillan, L. Ebony Boulware

**Affiliations:** Office of Research Initiatives, Duke University, Durham, NC, USA

**Keywords:** Consultation, research, support, navigation

## Abstract

Navigating the research domain at an academic medical center can be challenging, even for seasoned investigators. To address this, Duke University launched two initiatives: (1) a research navigation “hotline” to provide brief assistance with a variety of research questions; and (2) researcher onboarding and consultation, a one-to-one tailored offering to ensure that researchers are equipped to navigate research resources and processes effectively. The services are provided by the myRESEARCHnavigators (MRN) team, funded by Duke’s CTSA. The diverse scientific backgrounds of the six team members align well with those of the research community, allowing for a good match between the researcher and MRN team member. The MRN team answers approximately 30 questions per month, and has provided consultations to almost 400 researchers. Both services receive high satisfaction ratings (4 or 5 stars [out of 5 stars] given to 90% of hotline answers, and 99% of researcher onboarding/consultation sessions). As of July 2019, the School of Medicine has determined that the consultations are critical to their mission and have made them a requirement for new research faculty. The team will continue marketing both services to encourage adoption.

## Introduction

Navigating the research domain at an academic medical center can be challenging, even for seasoned investigators familiar with established systems and resources. The challenges multiply for junior faculty and trainees who, upon entering a new research environment, must adapt quickly to develop successful track-records of research funding and discovery. Senior faculty often provides the requisite guidance to help jumpstart these academic careers; however, clinical, teaching, or departmental demands often impede their ability to fill this mentoring gap [1–3]. Furthermore, senior faculty may not be as familiar with newer, innovative tools and resources being offered, as they may rely on tried-and-true methods that have led to their own personal success.

Increasingly, academic institutions are recognizing the need to more systematically equip newcomers to their research communities with the knowledge and tools to excel [4,5]. In 2016, for example, the Duke Clinical and Translational Science Institute (CTSI) surveyed over 250 faculty and ranked a research “navigator,” who could help to identify and engage research resources, as the third most useful proposed resource, after a self-service datamart and a researcher portal (unpublished data). Notably, over 60% of survey respondents ranked lower than full professor, highlighting a possible dearth of practical guidance available to more junior researchers.

To address the gap in services, Duke University launched two initiatives: (1) a research navigation “hotline” to provide quick answers to and brief assistance with a variety of research questions and problems; and (2) a researcher onboarding and consultation service, a one-to-one tailored offering to ensure that researchers are equipped to navigate research resources and processes effectively. The services and early outcomes are described.

## Materials and Methods

Duke University’s 12 schools and multiple institutes and centers house talented research faculty responsible for conducting a broad range of scientific research – from bench to bedside to population. Researchers hold appointments in various departments, and each department has its own method for onboarding, orienting, and supporting its faculty. Brief group orientation sessions are offered annually to new faculty by the university and School of Medicine (SOM), covering only general topics of broad interest (promotion and tenure, the history of Duke, etc.) and focusing largely on clinical science. Ultimately, to determine the best means for accomplishing their individual research goals, researchers needed to rely on advice from busy mentors and staff, scour newsletters, or learn through trial and error. The myRESEARCHnavigators (MRN) team, funded by Duke’s Clinical and Translational Science Award, sought to improve onboarding and ongoing guidance for researchers by providing high-quality support via two new services. Note that full-service descriptions can be found on the team’s website (http://researchinitiatives.duke.edu/).

### Navigation “Hotline” Service

The first service, a navigation “hotline,” was soft-launched in 2015 with two full-time staff members dedicated to answering questions related to basic, translational, and clinical research. One staff member was PhD-trained in experimental pathology, and the other was a project manager who had served in a support role in a central clinical research support office for 5 years. Both were well-versed in how to efficiently navigate the Duke environment. Researchers across the institution were able to access the hotline via a phone number or email address. While the service was successful and customers were very satisfied, the hotline was not widely publicized in the research community. During the service’s first 24 months, there were a total of 139 questions posed (5.8 per month), of which 42% were by faculty, 47% by staff, and 11% by others (e.g., trainees, external) [6].

In early 2017, the service was relaunched with new staff and heavily marketed. There are now six staff members, dedicating a portion of their time to this service (in addition to the consultation service described below). The total team effort for both services comprises 1.5 Full Time Effort (FTE) between the six staff, with the balance of their time spent engaging directly as members of various scientific teams and/or as administrative support. The team now includes three PhDs and three master’s trained staff in the fields of biochemistry, dermatology/HIV, molecular genetics, social science, data science, and exercise physiology. The backgrounds of the team members align well with those of the research community, allowing a good match between the person with the question and the team member providing support. All staff were hired from within the Duke research community, have several years of Duke-specific research experience, and had a demonstrated ability to provide high-quality customer service.

In addition to a retooled staffing model, the team engaged in heavy marketing via e-communications and presentations at many research faculty meetings. The service is now co-branded with the institution’s researcher “portal,” myRESEARCHhome [7–9]. The portal is branded as self-service support, and the research navigators are the hands-on helpers who can guide a user through various issues. By co-marketing the resources to the research community, the service has become more widely known and well used. In addition to co-marketing, the resources are functionally intertwined – the MRN team can be accessed via the “Get Help” widget in the portal. Users simply click on a question category (e.g., facilities and equipment) and submit a summary of the question, and then the question is routed immediately via email to all staff members. We have some anecdotal evidence to suggest that keeping the intake process short and simple reduces barriers to its use. During presentations where the service is described, faculty members who have used the hotline have voluntarily offered to their peers that it really is as easy as clicking a button and submitting your question.

### Researcher Onboarding and Consultation Service

As the new MRN team began fielding more questions from the research community, a new idea emerged – to avoid problems in initiating or carrying out research, there may be a need to better equip research community members *from the start of their time at Duke*. This was consistent with data (unpublished) from the 2016 CTSI survey of over 250 faculty, where respondents ranked this service as the third of eight most useful resources the CTSA could provide the research community with (trailing only a self-service datamart [#1] and self-service researcher portal [#2]). While many services and resources at Duke and within departments are available to researchers, they can be hard for researchers to find. In addition, researchers may not fully understand processes for activities such as submitting protocols for ethics review or preparing grants. This can lead to frustration and delays as last-minute issues are discovered and must be addressed.

As a result, the team set out to develop a researcher “onboarding” service to equip faculty with such requisite knowledge soon after their arrival at Duke. Prior to its launch, a senior staff member interviewed and pilot-onboarded approximately 15 recent faculty hires to understand how best to structure the program. Some of the greatest concerns expressed by new faculty included: (1) not knowing what resources, tools, and services are available to them, (2) who can help them with various tasks, and (3) why things get delayed and what to do about it. Given this pilot group feedback, the service would aim to: (1) help faculty know in advance about resources, offices, and process so they can anticipate the path forward and make good use of existing services, and (2) help set expectations for timelines and requirements, so they can be at lower risk of experiencing problems.

Duke University and its Schools of Medicine and Nursing hire hundreds of new faculty members each year, with most arriving in the summer. Hence, the MRN team launched the service in the summer of 2017. The service is structured as follows:

- Researchers are identified in several ways. All new research faculty in SOM are identified by central “new hire” lists (46%) and invited by the navigator team to attend a consultation. Researchers may also request the service on their own (12%), and others are referred by colleagues (33%) or other offices (9%).
- Faculty agreeing to the consultation are matched with the MRN team member who most closely aligns with their area of research.
- Prior to consultation, the research navigator investigates existing grants, published articles, etc., to understand the type of research in which the investigator engages.
- Sessions are one-to-one and last approximately 90 minutes. Typically, a departmental representative also attends to speak about unit-level processes and resources.
- Consultations are somewhat open-ended, starting with current questions the researcher may have, followed by a discussion of what he or she hopes to achieve in the ensuing 3–6 months. This increases the relevancy of the discussion and allows the navigator to address the most pressing items.
- Based on the exchange outlined above, the navigator gives an individualized consultation that covers the following topics:
  ○ Electronic resources the researcher will use, including the self-service myRESEARCHhome portal (http://myresearchhome.duke.edu)
  ○ Specific people who help to support the researcher, including the myRESEARCHnavigators team
  ○ Offices, processes, and resources the researcher will need or encounter given their upcoming activities
  ○ Data storage, management, and computing that will fit their needs
  ○ Researcher requirements and training
  ○ Funding sources and finding collaborators
  ○ Publication and writing resources
  ○ How to sign up for newsletters and discover seminars/events
- Additional consultation time can be scheduled as needed if the 90-minute session is not sufficient to address all of a researcher’s questions or concerns.
- The navigator sends a follow-up email within 1 week with a summary of what was discussed, including relevant links, contacts, and connections.
- The navigator reaches out after 3 months have passed: (1) to make sure there were no new issues and (2) to receive more long-term feedback about how helpful certain resources were to the investigator’s research progress.

An onboarding session takes the MRN staff member roughly 4.5 hours to complete. This includes the initial invitation, investigation of the researcher’s profile, an in-person consultation, and a 3-month follow-up.

## Results

**Fig. 1.**
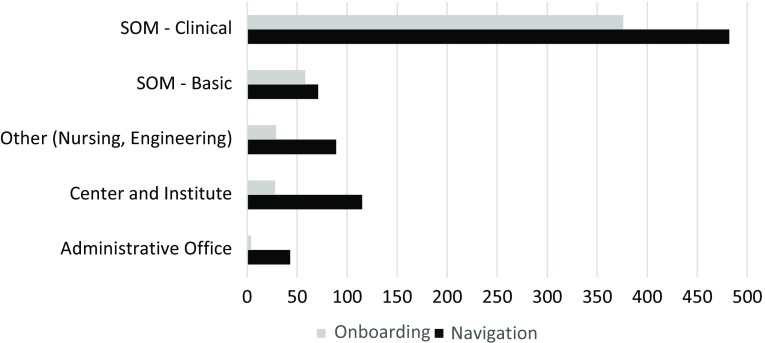
Services accessed by the School of Medicine (SOM).

**Fig. 2.**
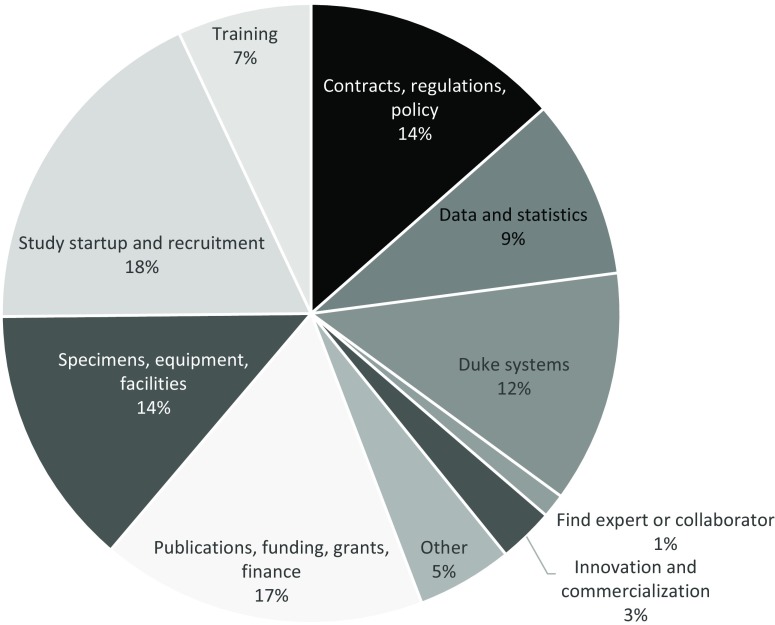
Number and types of questions handled by the navigation service (*N* = 800).

Both services are accessed by a variety of researchers from across the institution. Fig. 1 displays the use of each service by university school and unit. In the case of hotline service, users come with a broad scope of questions (note that IT-related questions are triaged to another service). A summary of the kinds of questions addressed by the MRN team since its inception in 2015 is provided in Fig. 2. Some common issues handled by navigators include: (1) researchers wanting to embark on a new methodology and to see if anyone else at Duke has experience, (2) what is the best resource at Duke or beyond for “X” (e.g., conducting an assay), (3) how to obtain data from patients for research, (4) whether or not there is a policy to guide “X” activity, and (5) help in understanding why their protocol has not yet received institutional approval, etc. The range of topics is incredibly broad and covers finding resources, interpreting policies and regulations, and assisting researchers in understanding the processes.

**Fig. 3.**
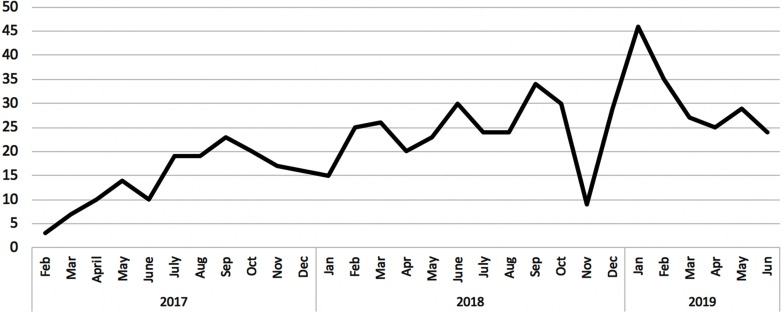
Volume of questions handled by the navigation service.

The number of issues addressed by the hotline each month has steadily increased, due largely to enhanced awareness of the service and awareness beginning at the time of onboarding (Fig. 3). While the original service saw just under six questions per month, the myRESEARCHnavigators team now addresses typically 25–30 questions per month, with much volatility given holidays and summer breaks. While this number may be relatively low, it is important to note that Duke’s office dedicated to clinical research support already had well-established helplines. So, these additional questions are those that either cannot be answered by that helpline or fall outside of their scope. The navigation hotline is simple in concept and delivery, and appears to meet the needs of most of its customers, as evidenced by its 90% four- or five-star ratings. In addition, 27% of the 479 users have returned to ask additional questions.

Researcher onboarding launched more recently has already been viewed very favorably by faculty, likely due to how the service is deeply tailored to the needs of each individual. As of June 2019, a total of 472 researchers have been offered the service and 84% accepted, and 382 have completed. Of those who completed the satisfaction survey (*N* = 152), 99% have given the service 4 (15%) or 5 (84%) stars. The uptake of the service fluctuates with the timing of new faculty hires, with high-volume months exceeding 25 researchers per month, and low-volume months hovering between 4 and 10. The service is required for SOM new faculty as of July 1, 2019.

The onboarding service is a primary avenue for increasing awareness of the navigation service. After the navigator establishes in-person rapport and trust with the researcher, and has an opportunity to demonstrate their ability to connect the researcher with relevant resources and people during their onboarding session, they are sometimes re-contacted via the hotline. To date, 21% of people who complete the onboarding session subsequently use the navigation service at least once. This level of reutilization is lower than might be expected, and could be due to several factors, including connecting well with unit-level assistance, finding direct assistance from the clinical research office, or not remembering to reach out to the MRN hotline service.

## Discussion

The simplicity of the navigation hotline service appears to be a strength; people inherently understand the concept of a hotline, and the intake process is as simple as submitting a question. Those who use the service provide high ratings, and more than a quarter have come back to ask additional questions. A challenge that remains for the hotline service is the lack of awareness that the service exists. This is somewhat ameliorated by the opportunity to talk about it in person, one-to-one, during onboarding.

The consultation and onboarding service derives its success from being tailored to the individual. By increasing the relevancy of the session, the investigator’s time is better spent. This service is considered valuable to individual researchers, and gained the attention of research leadership as a powerful means of reaching young faculty and trainees at the outset of their careers. High-touch, early guidance in the complicated research environment may reduce frustration and ultimately inspire them to engage in research activities. Administrative leaders are now encouraging new faculty outreach even before researchers arrive at the institution. These sessions, typically conducted via a brief phone call, can help the navigators identify sticking points, which can further smooth the transition as a faculty member transfers from another university. As the service becomes required for SOM faculty, the MRN team will emphasize the institution’s commitment to research integrity and quality, by discussing information about data management, laboratory culture, researcher responsibilities, etc.

Finally, a critical element that drives the success of the program is its people. All staff are experienced in research, so they understand the principles, the importance of quality, and the pressures many investigators face. This lends credibility to their interactions with researchers. Their experience and existing relationships at Duke, coupled with their ability to build rapport with others, have helped them earn reputation and trust from within the institution. This allows them to function as “central connectors” within the organization, informally linking people with one another [10]. Additionally, the use of portions of staff effort to fuel the model for these two services makes the program sustainable. The navigators are embedded in other parts of Duke’s research enterprise, ensuring that they remain in touch with real-world activities. By resourcing only portions of their effort, the program has potential for greater sustainability, and resiliency in the case of staff members leaving.

Looking ahead, the goal for these two services is to maintain existing format, build on successes, and increase awareness. Ultimately, it will be important to understand if either of these services makes a difference in the quality and efficiency of research conduct. Are those who take part in the services more likely to receive grant funding earlier in their tenure at Duke? Do those who seek assistance from the navigators have fewer delays with institutional approval processes? To answer these and other questions, we will continue to track engagement with the services, and rely on institutional data for related outcomes, to consider whether that engagement can predict success. Institutional data could include dates that certain approval milestones are met, utilization of core/shared resources, research dollars awarded, number of co-collaborators, etc. Careful consideration will need to be given to confounding variables, as a true control group for this analysis would be difficult to formulate. Similarly, a measure of learning is not included in the services, so it is difficult to know whether the researchers retain the information that is shared.

Ultimately, the services described here are likely best suited for institutions that are complex, with several schools, and having many resources and services. Many of the issues that the team helps navigate are a function of the decentralized nature of service offerings, as well as their quantity.

## Previous Presentations

An early report of these services was shared, in part, during a poster session of the 2018 Conference of the Association for Clinical and Translational Science.
